# Informant characteristics are associated with the Clinical Dementia Rating Sum of Boxes scores in the Alzheimer’s Disease patients participating in the National Alzheimer’s Coordinating Center Uniform Data Set

**DOI:** 10.1002/alz.090457

**Published:** 2025-01-03

**Authors:** Juan‐Camilo Vargas‐González, Antonella Santuccione, Laura Castro, Maria Teresa Ferretti, Maria Carmela Tartaglia

**Affiliations:** ^1^ University Health Network, Toronto, ON Canada; ^2^ Euresearch, Bern Switzerland; ^3^ Women’s Brain Project, Guntershausen Switzerland; ^4^ Women’s Brain Project, Bottighofen Switzerland; ^5^ Karolinska Institutet, Stockholm Sweden; ^6^ Division of Neurology, Krembil Neuroscience Centre, Toronto Western Hospital, University Health Network Memory Clinic, Toronto, ON Canada

## Abstract

**Background:**

The Clinical Dementia Rating Sum of Boxes (CDR‐SB) is a widely used measurement score to stage dementia severity; it has become one of the most common outcome measurements in Alzheimer’s Disease (AD) research. The CDR‐SB requires an informant to provide information to stage a patient’s dementia severity. The effect of the informant’s characteristics including sex and relationship with patients on the CDR‐SB is unknown. We aimed to evaluate the effect of the informant’s sex and relationship to the patient on the CDR‐SB scores in patients with Alzheimer’s Disease with mild cognitive impairment or dementia included in the National Alzheimer’s Coordinating Center Uniform Data Set (NACC‐UDS).

**Method:**

We included all participants from the NACC‐UDS that had AD as the principal diagnosis, with information about the Mini‐Mental State Examination or Montreal Cognitive Assessment scores, and informant sex and relationship to patient; we also analyzed the possible interaction between these characteristics, and the outcome was the CDR‐SB. We performed a conditional growth model using multilevel linear regression analysis.

**Result:**

We included data from 20636 participants, totalling 47727 visits with a median of 2 (1‐4) visits. Patients' age at the initial visit was 74.0±9.4 years and 54.1% were females. Informant characteristics were mean 66.2±13.2 years of age, 69.1% were females, and the relationship to patients was 60.5% spouse or partner, 26.7% children and 12.8% other relation. The CDR‐SB scores were 0.20 (CI 95%: 0.11 to 0.29) higher when the informant was female. When comparing to informant’s relationship with the baseline being spouse or partner, the CDR‐SB was 0.39 (CI 95%: 0.25 to 0.53) higher when the informant was the child of the patient and 0.18 (CI 95%: ‐0.35 to ‐0.01) lower if other relationship. The only significant interaction was between informant relationships other than spouse or child and female patients where the scores were 0.24 (CI95%: 0.03 to 0.46) higher.

**Conclusion:**

We found that the CDR‐SB scores are significantly modified according to informant characteristics in the NACC‐UDS patients with AD diagnosis. These results are clinically relevant because informant characteristics should not affect AD patient staging, and future research should evaluate how to manage these confounding variables.

